# Predicting the Potential Suitable Climate for Coconut (*Cocos nucifera* L.) Cultivation in India under Climate Change Scenarios Using the MaxEnt Model

**DOI:** 10.3390/plants11060731

**Published:** 2022-03-09

**Authors:** Kukkehalli Balachandra Hebbar, Pulloott Sukumar Abhin, Veliyathukudy Sanjo Jose, Poonchalikundil Neethu, Arya Santhosh, Sandip Shil, P. V. Vara Prasad

**Affiliations:** 1 Indian Council of Agricultural Research—Central Plantation Crops Research Institute, Kasaragod 671124, Kerala, India; abhinsukumarp@gmail.com (P.S.A.); neethupsankaran@gmail.com (P.N.); aryaaromal36@gmail.com (A.S.); 2 Forest Research Institute, Dehradun 248001, Uttarakhand, India; sanjojosev@gmail.com; 3 Indian Council of Agricultural Research—Central Plantation Crops Research Institute Research Centre, Mohit Nagar 735101, West Bengal, India; sandip.iasri@gmail.com; 4 Sustainable Intensification Innovation Lab, Kansas State University, Manhattan, KS 66506, USA; vara@ksu.edu

**Keywords:** coconut, high temperature, prediction, MaxEnt, vulnerability, climate change

## Abstract

Climate change and climate variability are projected to alter the geographic suitability of lands for crop cultivation. Early awareness of the future climate of the current cultivation areas for a perennial tree crop like coconut is needed for its adaptation and sustainable cultivation in vulnerable areas. We analyzed coconut’s vulnerability to climate change in India, based on climate projections for the 2050s and the 2070s under two Representative Concentration Pathways (RCPs): 4.5 and 8.5. Based on the current cultivation regions and climate change predictions from seven ensembles of Global Circulation Models, we predict changes in relative climatic suitability for coconut cultivation using the MaxEnt model. Bioclimatic variables Bio 4 (temperature seasonality, 34.4%) and Bio 7 (temperature annual range, 28.7%) together contribute 63.1%, which along with Bio 15 (precipitation seasonality, 8.6%) determined 71.7% of the climate suitability for coconuts in India. The model projected that some current coconut cultivation producing areas will become unsuitable (plains of South interior Karnataka and Tamil Nadu) requiring crop change, while other areas will require adaptations in genotypic or agronomic management (east coast and the south interior plains), and yet in others, the climatic suitability for growing coconut will increase (west coast). The findings suggest the need for adaptation strategies so as to ensure sustainable cultivation of coconut at least in presently cultivated areas.

## 1. Introduction

Coconut (*Cocos nucifera* L.) is an environmentally friendly smallholder palm of the tropical environment, cultivated in more than 94 countries in the world over 11.99 M ha, producing 67.04 billion nuts with a productivity of 5592 nuts ha^−1^ [1]. Indonesia is the largest coconut-producing country followed by the Philippines, while India, with 2.1 million ha and 2.73 million t copra, occupies third place in area and second place in production. Around 80 million people depend directly on coconut for their livelihood [2]. The coconut industry, which traditionally relied upon copra and coconut oil, and to some extent coir, is experiencing a tremendous transformation towards product diversification, high value product development, by-product utilization, and more importantly, is now being used as health drink [3]. Of late, the nutraceutical and functional food properties of tender coconut water, virgin coconut oil [4], and inflorescence sap [5,6] are being harnessed for a diversity of health products and preventive medicine applications [7,8,9]. The health benefits of coconut are driving its sales. As a result of this, since 2000, a steady annual increment has been seen for coconut and coconut products and this is predicted to continue increasing further [10].

To strike a balance between future demand and supply, production has to be augmented either through increased productivity or through cultivating a larger area. The global cultivation area of coconut clearly indicates that no significant area expansion has taken place during the 2010 to 2015 period. Meanwhile, during the last decade, coconut growing regions experienced frequent severe weather events like drought and flood and numerous pests and diseases infestations, as a result productivity declined, and global coconut production stagnated [11]. Hence, it may be a great challenge to increase the expected production unless adoptive measures against the predicted threat of climate change are addressed.

Climate variables such as temperature, precipitation, and salinity have enormous impacts on the growth and development of coconut as in other species, and these factors had restricted its cultivation to southern geographical regions of India [12,13,14,15]. These variables alter the physiology, phenology, behavior, and ecological interactions of the crops [16,17,18] and affect the production faster than expected [19]. High temperature, to a large extent, had offset the otherwise positive effect of the rising atmospheric CO_2_ on coconut seedlings, which occurs in many C_3_ crops [20]. This suggests that climate change variables must be assessed together to ascertain how a changing climate will impact coconut [21]. The type of combinatorial experiments that study the effects of both warming and elevated CO_2_ on coconut at different stages of growth are very much limited. Coconut grows well in north India, but when the summer temperature goes up (T_max_ > 40 °C) under low humidity nut production is severely dented. Pollen germination on stigma [22] and pollen tube growth through style [23] in coconut is highly sensitive to high temperature resulting in poor fertilization and nut set. Similar to high temperature, water limitation is another common limitation in coconut ecosystems; more than 60% of the crop is grown under rainfed condition. The interaction effects of rising CO_2_, warming, and water deficit in coconut is not well studied, but is well studied in other perennials like grassland [24,25] or cocoa [26]. The lack of sufficient data on the response of coconut to climatic variables led us to use the correlative model MaxEnt to assess the coconut suitability/unsuitability of a region.

In order to predict resulting changes in the relative climatic suitability of crop-growing regions under future climate scenarios, MaxEnt is widely used and can run with presence data alone [27]. MaxEnt is considered to be the best method among the species distribution modeling (SDM) techniques [27,28] due to its higher success rate and excellent results even with a low sample size [29]. It is an important aid in understanding the influence of climate change on species distributions [30,31,32]. MaxEnt was used to predict the change in climate of some of the plantation growing areas like areas growing cocoa in African countries [33,34], coffee in Zimbabwe [35], and other agricultural crops [36,37,38]. Despite the important role of coconut in safeguarding the livelihood of millions of people in the south Indian region, the literature shows that there has been little research into the future climate suitability of the region for coconut cultivation. The present study aims to evaluate the potential impacts of climate change on the suitability of a habitat for coconut cultivation and fill this key research gap. We used the MaxEnt model with the kuenm framework in R software [39]. The specific objectives of this study were to (a) determine the potential impacts of environmental variables on coconut cultivation; (b) model the current and future suitability for coconut cultivation under two climate scenarios (RCP 4.5 and RCP 8.5) for the years 2050 and 2070; and (c) identify the potential changes in the suitability of the land for coconut cultivation. We also suggest adaptation measures to reduce the vulnerability of coconut to the projected changes.

## 2. Methods

### 2.1. Study Area

**Figure 1:**
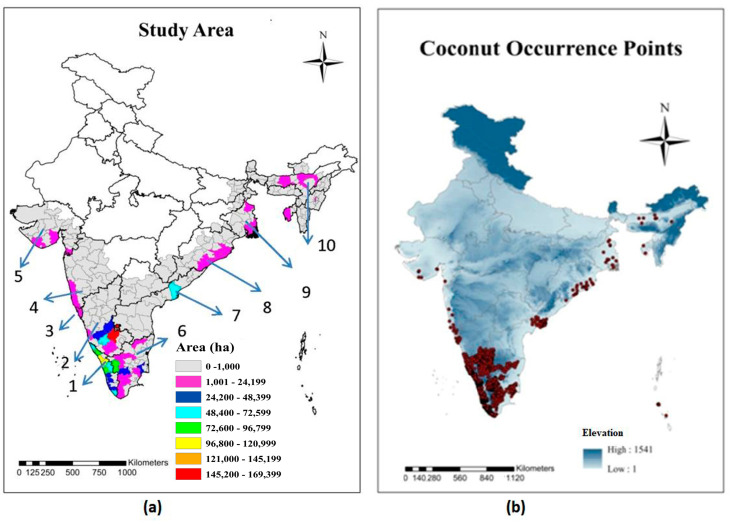
(**a**) Map showing the spatial distribution of coconut in different regions of India. State-wise and district-wise area data 2018-19 obtained from https://coconutboard.nic.in/Statistics.aspx, accessed on 7 January 2021 Numbers on the map show the coconut growing states viz. Kerala (1), Karnataka (2), Goa (3), Maharashtra (4), Gujarat (5), Tamil Nadu (6), Andhra Pradesh (7), Odisha (8), West Bengal (9), and Assam (10); and (**b**) the red point indicates the coconut occurrence points used in model running.

The coconut palm in India is grown under varying climatic and soil conditions mostly between 8°4′ N and 20° N latitudes. The study area and coconut occurrence points are shown in Figure 1. Major coconut growing states and their district-wise area and climate and soil characteristics are presented in Table 1. During the 1960s, the west coast of India, i.e., Kerala and coastal Karnataka, were the traditional coconut cultivation areas. Out of the total area, the major share of nearly 70% was in Kerala followed by 13.65% in Karnataka, only 7.6% in TN (Tamil Nadu), and 4.8% in AP (Andhra Pradesh). Over the years, cultivation has spread to the plains of Karnataka and TN, resulting in increase in the Karnataka and TN share to 28% and 20.3%, respectively, with only a marginal increase in AP (5.2%) and a sharp decline in the Kerala share to 35% (CDB; https://www.coconutboard.gov.in, accessed on 22 June 2021). The west coast, with its high rainfall (annual rainfall is >2000 mm) and moderate temperature (Tmax, maximum temperature of 34 to 36 °C), is ideal for coconut cultivation. However, on the east coast, the rainfall is low (around 1000 to 1200 mm) and Tmax reaches a maximum of 40 to 42 °C in some of the coconut growing regions.

### 2.2. Coconut Occurrence Data

The data on major coconut growing states and districts was sourced from the Coconut Development Board (CDB; https://www.coconutboard.gov.in, accessed on 7 January 2021) website. From the list, the districts with a large area under coconut cultivation were selected for the study. From each of these districts, the names and areas of the village with extensive coconut cultivation were obtained from the agriculture/horticulture officer of the respective district. In addition to these, the district and village level data for the state of Karnataka was sourced from the crop survey website (www.cropsurvey.karnataka.gov.in, accessed on 23 February 2021). In India, a village is a clustered human settlement or community, larger than a hamlet but smaller than a town, with a population typically ranging from a few hundred to a few thousand. Villages are the smallest unit for which the coconut cropped areas and production records are maintained by the agricultural or horticultural offices. Through this process, nearly 3000 coconut occurrence points were collected from different coconut growing regions, followed by manual verification using Google Earth map. The Google Earth platform provides high-resolution images of coconut orchards and it is, therefore, suitable for identifying coconut cultivation areas. These data, along with the point data collected using Global Positioning System (GPS) in previous studies, constituted the occurrence points (Figure 1b). To reduce the issue of spatial sampling biases caused by multiple autocorrelated locations, the coconut occurrence points were spatially rarefied at 5 km using the SDM Toolbox 2.0 [40] in ArcGIS v. 10 (Registration Number EFL431708926). The final coconut occurrence dataset used for building SDMs included 1008 occurrence records. Figure 1b presents the final coconut records utilized for the modeling exercise.

### 2.3. Selection of Environmental Variables

As environmental predictors, we used 19 bioclimatic variables (Table 2) from Paleoclim.org [41] for historical (1979–2013) and current climate (baseline) data and from the World Clim.v1.4 database (http://www.worldclim.org/download, accessed on 15 March 2020) [42] for the future climate data for the 2050s (average for 2041–2060) and 2070s (average for 2061–2080). Variables representing the two future scenarios ((representative concentration pathway RCP 4.5 (intermediate scenario) and RCP 8.5 (very high emission scenario)) were an ensemble of 7 GCM Models (BCC-CSM1-1, GFDL-CM3, HadGEM2-ES, MIROC5, MIROC-CHEM, MIROC-ESM, NorESM1-M), because of their good predictive ability of climate for India [43,44]. Predictors were obtained at two-and-a-half-minute spatial resolution (approximately 5 km^2^ per pixel), which is an adequate resolution for ecological niche models based only on climate variables [29].

It is recommended to include non-climatic variables to enhance the predictive performance of SDMs [45,46]. Hence, we included soil and two topographical variables elevation and land cover (Table 2), which have a considerable influence on coconut cultivation in India. Soil data were downloaded from FAO (http://www.fao.org/soils-portal/data-hub/soil-maps-anddatabases/harmonized-world-soil-database-v12/en/, accessed on 22 May 2020), and elevation data were sourced from the shuttle radar topography mission dataset (https://cgiarcsi.community/data/srtm-90m-digital-elevation-database-v4-1/, accessed 22 May 2020). The source of land cover data was Glob Cover 2009 (http://due.esrin.esa.int/page_globcover.php, accessed on 22 May 2020). All the data were converted into two-and-half minute spatial resolutions (approximately 5 km^2^ per pixel), the same as that of climate variables.

To select a distinct set of variables that contributed the most to the models, we used the correlation analysis of the SDM Toolbox 2.0 by eliminating one variable per pair with correlations of (*r* > 0.85) (Table 3). Seven highly correlated climate variables were eliminated and the remaining 12 bioclimatic variables, along with 2 topographical variables and soil were selected for the model calibration.

### 2.4. Model

The climate suitability for the cultivation of coconut under the future climate was studied using MaxEnt 3.4.1 [27]. This software is based upon the maximum entropy principle, which is freely available at (url:http://biodiversityinformatics.amnh.org/open_source/MaxEnt/, accessed on 21 November 2021), and holds anything with the maximum entropy closest to its real state under known conditions [47]. The MaxEnt model is used to estimate the target probability distribution by finding the probability distribution of the maximum entropy (i.e., that which is most spread out or closest to uniform) that is subject to a set of constraints that represent our incomplete information regarding the target distribution [48,49].

### 2.5. Model Calibration

The kuenm R package is used for the detailed calibration of the MaxEnt model, its selection, final model creation, and evaluation [39]. The occurrence data is split into 75–25 subsets for model calibration and internal testing, respectively, using the kuenm_occ_split function. The kuenm varComb function was used to generate 16 sets of environmental predictors for calibration, with the minimum number of variables taken as 14. The model chose variable predictor set 6 (which excluded Bio 5) for final model running. The variables used for the final model running with their description are listed in Table 2, and a detailed description of each variable is available in O’Donnell and Ignizio [50]. We created 2480 candidate models by combining 16 sets of environmental predictors, 5 values of regularization multiplier (0.1, 0.2, 0.3, 0.4, 0.5), and 31 combinations of feature classes (l, q, p, t, h, lq, lp, lt, lh, qp, qt, qh, pt, ph, th, lqp, lqt, lqh, lpt, lph, lth, qpt, qph, qth, pth, lqpt, lqph, lqth, lpth, qpth, lqpth). The candidate model performance was evaluated based on significance (partial ROC, with 500 iterations and 50% of data for bootstrapping), omission rates (E = 5%), and model complexity (AICc). Final model selected is the one with the lowest omission rate and delta AICc values of ≤2. After the creation of the final model with the parameter sets selected as best, the model projections were made for RCP 4.5 and 8.5 for the years 2050 and 2070 using the kuenm_mod function. The free extrapolation transfer was selected for future projections.

### 2.6. Model Evaluation

The area under the curve (AUC) or receiver operating characteristics (ROC) was used as the MaxEnt predictive performance metric under the ROC curve. The AUC was an effective threshold independent index that could evaluate a model’s ability to discriminate presence from absence (or background) [51]. The MaxEnt output provides an AUC or sensitivity vs 1-specificity graph, which describes the accuracy and fit of the predicted model. An AUC value of 0.5 indicated that the model performance was no better than random, while values close to 1.0 indicated better model performance [51]. The closer the AUC was to 1, the better the model performance.

### 2.7. Threshold Selection

The logistic output format ranging from 0 (unsuitable) to 1 (maximum suitability) was adopted for the model results, which indicates climate suitability for the cultivation of coconut (probability of presence) [52]. Binary model predictions from each scenario were overlapped in Arc-GIS v. 10.0 to map the current climatic range and potential future expansion and contraction. The binary suitable/unsuitable area was calculated for each scenario (current, RCP 4.5, and RCP 8.5), using ‘maximum training sensitivity plus specificity’ as the logistic threshold cut off value [53] to give an objective numerical overview of potential climatic suitability contraction and expansion. Maximizing the sum of the specificity and sensitivity logistic threshold was used to differentiate between presences and absences, as is recommended for models that use presence-only and background data (e.g., MaxEnt). It minimizes the mean of the error rate for positive observations and the error rate for negative observations [54].

The present and future suitability maps produced by the model ranged from 0 to 1. These maps were reclassified into five suitability classes, i.e., ‘Barely’ (0–0.05), ‘Very Low Suitability’ (0.05–0.25), ‘Low Suitability’ (0.25–0.45), ‘Moderate Suitability’ (0.45–0.65), ‘High Suitability’ (0.65–0.85), and ‘Very High Suitability’ (0.85–1.0). Binary rasters were used to analyze the predicted contraction and expansion areas using the SDM toolbox 2.0. ‘Barely’ and ‘Very Low Suitability’ classes were considered to be unsuitable categories as per the threshold value, and remaining classifications are considered to be suitable categories.

## 3. Results

### 3.1. Outcome of Model Calibration

The candidate models (2480) generated were statistically significant and better than null expectations (i.e., predictions from the models were in conformity with testing occurrence data more frequently than would be expected by random association of points and prediction of that areal extent) (Table 4).

**Figure 2:**
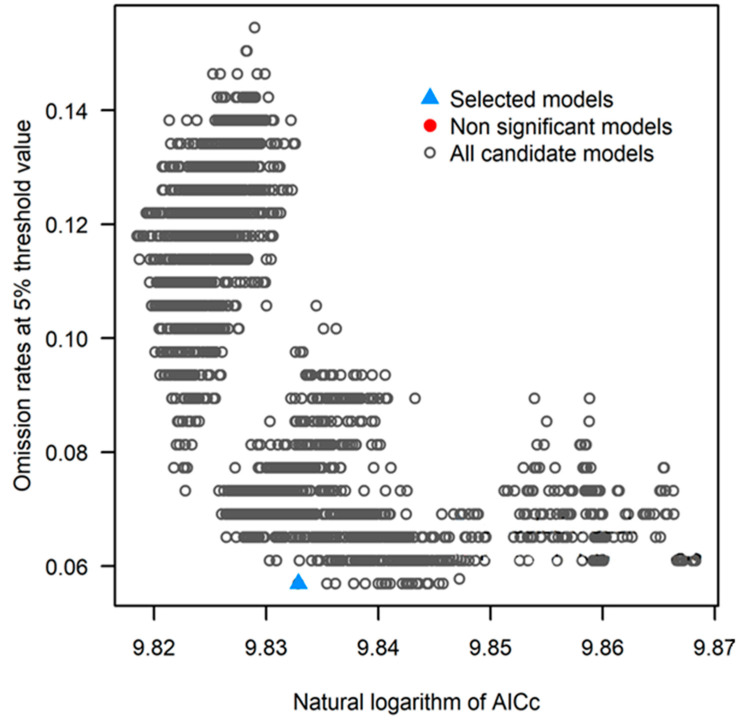
Omission rates at 5% and AICc values for all, non-significant, and selected ‘best’ candidate models for coconut. Models were selected based on statistical significance, omission rates, and AICc values.

Of the candidate models, none of the models met the omission rate criterion; however, two models had delta AICc values ≤2. Applying the three evaluation criteria together, one candidate model M_0.1_F_qp met the full suite of selection criteria for coconut (Figure 2).

### 3.2. Model Evaluation

**Figure 3:**
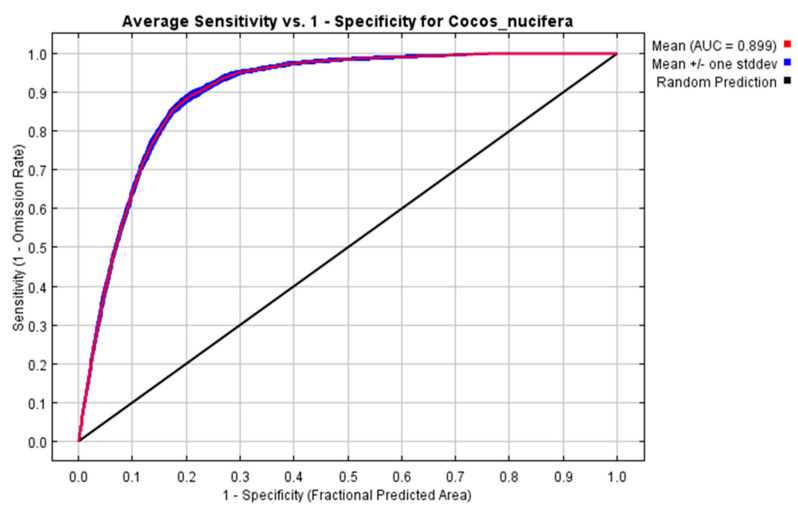
The receiver operating characteristic (ROC) curve. The values shown are the average of 10 replications.

The area under the curve (AUC) or receiver operating characteristics (ROC) was used to evaluate the MaxEnt model performance. The results of the model provided satisfactory output for the climate suitability prediction for coconut (AUC = 0.899 ± 0.002) (Figure 3).

### 3.3. Predictor Variable Influence

The importance level of predictor variables utilized in the MaxEnt model were identified and highlighted in bold in Table 2. The potential distribution of coconut is more strongly influenced by bioclimatic factors than soil and topographic factors. Bio 4 (temperature seasonality, 34.4%) had the greatest influence followed by Bio 7 (temperature annual range, 28.7%). These two together contributed 63.1%, which along with Bio 15 (precipitation seasonality, 8.6%), determined 71.7% of climate suitability for coconut in India (Table 2). Bio 3 (isothermality), Bio 6 (min. temperature of coldest month), and Bio 14 (precipitation of driest month) are the other major environmental factors and had an influence of 5.6%, 4.4%, and 4.2% respectively. Topographical parameters like elevation, land cover, and soil did not have a marked influence on model prediction (<4%).

**Figure 4:**
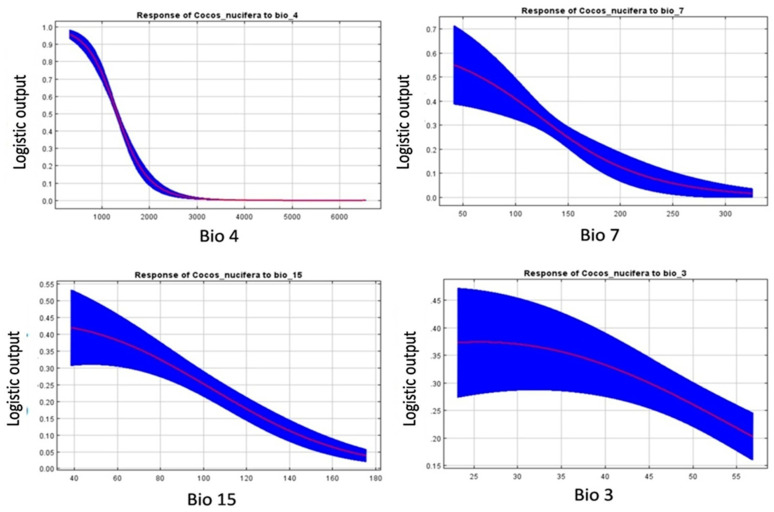
The Response Curves show how each environmental variable affects the MaxEnt prediction.

The graph (Figure 4) depicts the quadratic relationship between bio variables and climate suitability. Temperature seasonality (Bio 4) was found to have the highest climate suitability at 3% and the relationship was inverse. With the increase in temperature seasonality, the climate suitability of coconut is rapidly decreasing. It ranges from value 3 to 19 (greater than the 0.25-threshold value). Similarly, for temperature annual range (Bio 7), the suitability was the highest at around 3 °C and gradually decreased (range 3 to 15 °C). That is, the regions with low difference between the maximum temperature of the warmest month and the minimum temperature of the coldest month were more suitable for coconut cultivation. Precipitation seasonality (coefficient of variation) ranging from 4% to 10% (>0.25 threshold value), showed higher suitability for coconut cultivation (Figure 4).

### 3.4. Regional Changes in Predictor Variables

Table 5 shows the model simulated changes in predictor variables under the future scenarios RCP 4.5 and RCP 8.5 of 2050 and 2070 from the current scenarios of coconut cultivation regions. The mean annual temperature over the plantation growing regions of India is 25.64 °C under the present climate, and the model had predicted rise in temperature in all of the scenarios. RCP 4.5 and RCP 8.5 had projected an increase of 1.73 °C and 2.21 °C by the 2050s and 2.1 °C and 3.3 °C during the 2070s, respectively (Table 5).

Bio 4 variability, the bioclimatic variable, was at its lowest for the west coast (Kerala followed by Karnataka) compared with the east coast (TN and AP) (Table 5). The variability was 1.59%, 2.54%, 2.91%, and 3.39% for Kerala, Karnataka, TN, and AP respectively for RCP 8.5, 2070. The trend was similar during the 2050s for both the scenarios, but the increase was relatively less. The variability, however, was at its lowest with RCP 4.5, 2070 (0.88%, 0.99%, 1.46%, 2.62% for Kerala, Karnataka, TN, and AP respectively). Similar to Bio 4, the range of extreme temperature conditions (Bio 7) showed a higher fluctuation on the east coast ((2.38 °C for AP (RCP 4.5, 2050) and 1.77 °C for TN (RCP 8.5, 2070)) compared with the west coast ((1.37 °C for Karnataka (RCP 8.5, 2070) and 1.16 °C for Kerala (RCP 4.5, 2050)).

Precipitation seasonality (Bio 15) showed a relatively high increase under the future scenarios of AP and TN with a relatively smaller increase for Karnataka (Table 5). The increase was high for RCP 8.5, 2070. While it was at its lowest for RCP 4.5, 2070. Kerala, under all of the scenarios, showed marginal decline in precipitation seasonality.

### 3.5. Current and Future Projections

**Figure 5:**
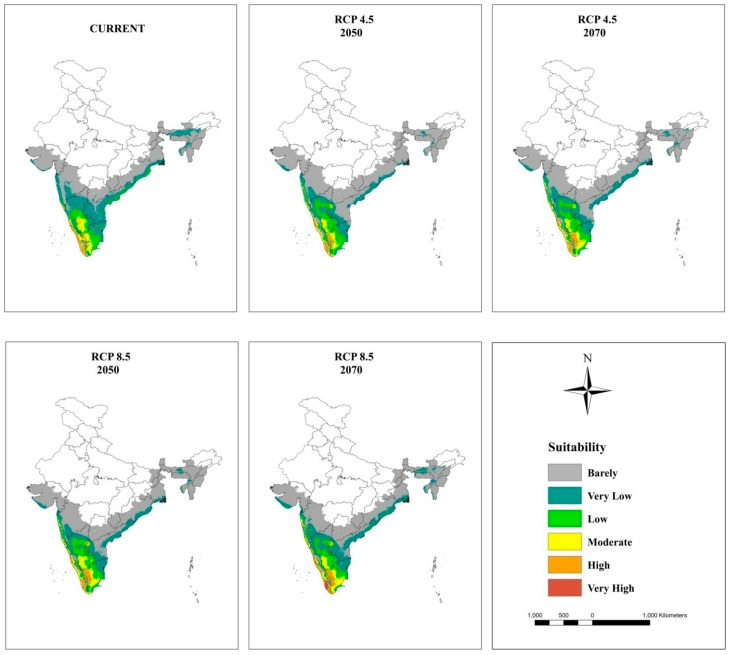
Climatically suitable areas for coconut production in India under the current and future climates of the 2050s and 2070s for RCP 4.5 and RCP 8.5 as modeled by MaxEnt.

**Figure 6:**
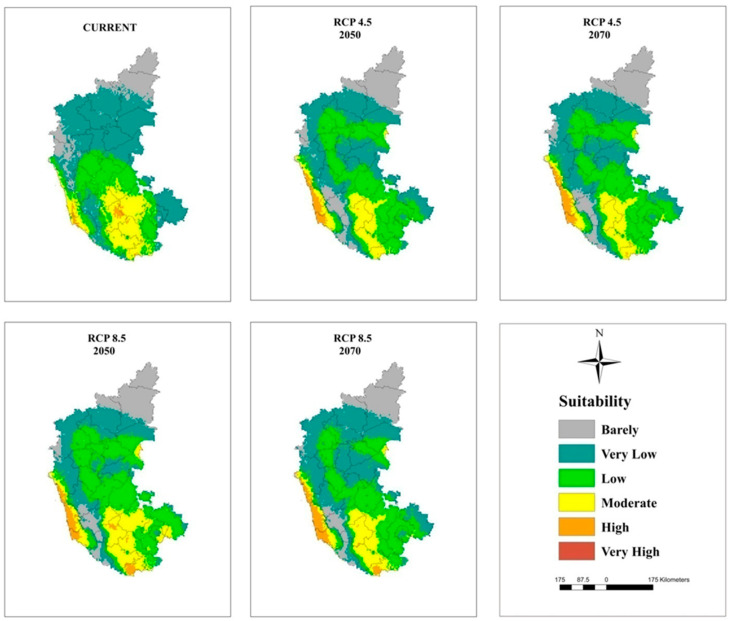
Climatically suitable areas for coconut production in Karnataka under the current and future climates of the 2050s and 2070s for RCP 4.5 and RCP 8.5 as modeled by MaxEnt.

**Figure 7:**
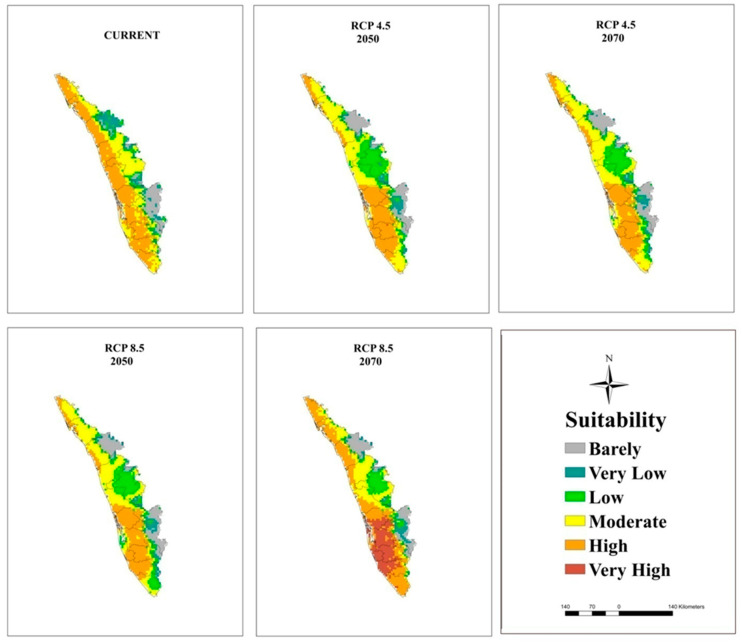
Climatically suitable areas for coconut production in Kerala under the current and future climates of the 2050s and 2070s for RCP 4.5 and RCP 8.5 as modeled by MaxEnt.

**Figure 8:**
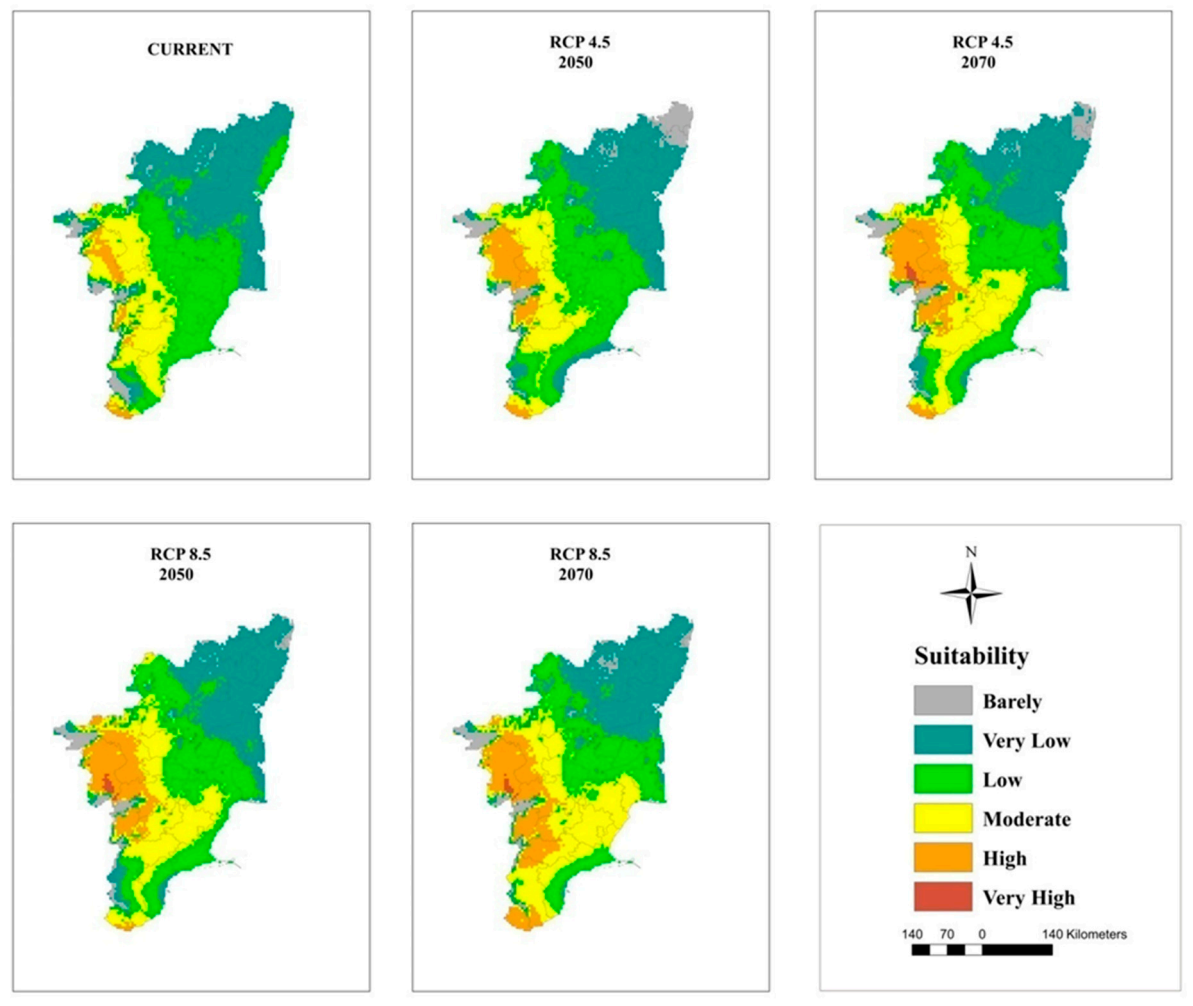
Climatically suitable areas for coconut production in Tamil Nadu under the current and future climates of the 2050s and 2070s for RCP 4.5 and RCP 8.5 as modeled by MaxEnt.

The maps of potential distribution of coconut at a national level (in India) under the current climate and RCP 4.5 and 8.5 for 2050 and 2070 generated by the MaxEnt model are shown in Figure 5 and Table 6. The model projected that an area of 209,374 km^2^ is suitable for coconut cultivation under the current climate, which is 15% of the study area in India. However, only 7% of this area is on the west coast and in South Interior Karnataka (Figure 6), Kerala (Figure 7), and TN (Figure 8) has moderate to high suitability, while the east coast and northeast have low to very low suitability. The area suitable for coconut cultivation in India has dropped to 11.6%, 12.1%, 12.4%, and 12% for RCP 4.5, 2050, RCP 4.5, 2070, RCP 8.5, 2050, and RCP 8.5, 2070, respectively (Table 6). The maximum decline was seen in the south interior regions.

### 3.6. Changes in Coconut Habitat Suitability

**Figure 9:**
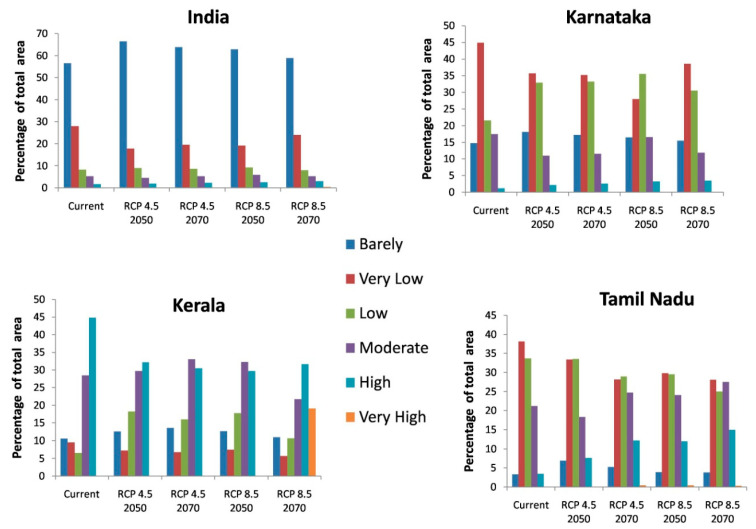
Percentage of total predicted area under different classes for the current and future climate of the study area of India and the regional levels of Karnataka, Kerala, and Tamil Nadu.

In general, the habitat suitability for coconut cultivation at a national level (in India) remained more or less constant at the future climates of RCP 4.5 and 8.5 of the 2050s and 2070s (Figure 9, Table 6). It is roughly 15% with 8% in the low category, 5% in the moderate category, and 2% in the high suitability category (Figure 5 and Figure 9). However, there is a shift in climate suitability category across the coconut cultivation regions of India under the future climate. Karnataka, which has, at present, 22% in the low category, 18% in the moderate category, and 1% in the high suitability category may shift to have 31% to 33% in the low category, 11% to 12% in the moderate category, and 2% to 3% in the high category across the scenarios (Figure 9). The west coast has high suitability, while the south interior moves to the low suitability category (Figure 6). Kerala, on the other hand, which has large area in high suitability category (45%) at current climate will shift to have low suitability and low suitability areas will increase from the current 7% to 16% to 18% under the future climate (Figure 9). South Interior Tamil Nadu (Figure 8) would have 8% to 12% high suitability areas under future scenarios from the present 4% (Figure 9). Area expansion is mostly seen in the barely suitable category, which is not considered for climate suitable area calculation.

## 4. Discussion

This study was the first to explore the impacts of global climate change on the geographical range and environmental suitability of the habitat of the coconut in India using MaxEnt modeling. As coconut is grown across different agro-ecological zones of India, evaluating the impacts of climate change scenarios on the potential cultivable area will be helpful in understanding the relationships between coconut niches and the corresponding environment, identifying priority cultivation areas and planning adaptation strategies [55,56,57]. Species distribution models like MaxEnt are extensively used to predict the change in climate of some of the plantation growing areas like areas growing cocoa in African countries [33,34], coffee in Zimbabwe [35], and other agricultural crops [36,37,38]. In our study, for model calibration, we could generate 2480 candidate models involving environmental predictors and regularization multiplier and feature classes using the kuenm-R package and select the best candidate model (M_0.1_F_qp) for prediction based on significance, omission rates, and complexity [39]. The results show that the MaxEnt model prediction for coconut, having the mean AUC values of 0.899 + 0.002 and indicating the model prediction, comes under the excellent category, which is consistent with previous studies [29,57,58].

The MaxEnt model showed that future coconut cultivation is mostly determined by bioclimatic variables, while the effects of topographical and soil variables were rather small. In 29 African palm species, the belief that the climate is the most important factor determining palm distribution, and that habitat and human intervention are not, was extensively studied and reported [59,60]. As in our studies, temperature seasonality, which contributed most to the habitat suitability along with cold temperatures, was found to constrain palm species cultivated [61]. In India, the majority of coconut cultivation area is within an elevation of 600 m, and the topographic effect on palms is generally seen at higher elevation of around 2500 m [62]. Though the contribution of soil, a topographic factor, is rather small (0.4%); however, its inclusion in the model made the prediction more accurate as seen in other species [63], otherwise, the model would have predicted more suitable area than currently exists for the actual current coconut distribution. Thus, as seen in previous studies, we have confirmed the dominant role of the climate in the natural cultivation of coconut [29,58].

As coconut is a tropical plant, its cultivation is mainly determined by the temperature. Optimal temperature for its growth and yield is 27 °C ± 5 °C and optimum humidity of >60%. The plant grows well up to an elevation of 600 m above MSL. However, near the equator, productive coconut orchards can be established up to an elevation of about 1000 m above MSL, providing that temperature is not a limitation. A well-distributed rainfall of about 200 cm per year is the best for proper growth and higher yield [64,65,66,67]. South India, comprising the states of Kerala, Karnataka, Tamil Nadu, and Andhra Pradesh, occupies >90% of the total coconut cultivation area in the country. All along the west coast is relatively ideal for coconut cultivation where rainfall is >200 cm, humidity is high, and T_max_ reaches as high as 36 °C during the summer (March, April, May). In the south interior, where rainfall is around 60 cm, T_max_ reaches as high as 40 °C, and summer humidity is low, coconut grows well wherever there are irrigation facilities. However, in this region, large areas are under rainfed conditions. On the other hand, in the eastern region mostly, coconut is cultivated under irrigation conditions where the rainfall is around 100 cm and T_max_ reaches as high as 43 °C. A high temperature (T_max_ > 34 °C) decreases the photosynthesis of the coconut seedlings [68] during the reproductive stage. This affects the progamic phase, i.e., causes poor pollen germination [22] and restricts pollen tube growth through style [23]. As a result, there was poor fertilization and nut set. In the field condition, it was observed that a prevailing high temperature (T_max_ ≥ 33 °C) during the first three months of inflorescence opening severely reduced the nut set of an inflorescence during the summer months both in India and Sri Lanka [69,70,71]. Furthermore, when water is limited, as is common in coconut ecosystems because more than 60% of the water supply is rainfed, the interaction effects of rising CO_2_ and warming with water deficit are not known. Therefore, for a crop like coconut, temperature, water, CO_2,_ and their interactions are the important climatic factors determining the suitability of a given region.

The MaxEnt model has predicted that the climate of the region between 8°4′ north and 20° south of the equator under current the conditions is ideal for coconut palm in India, consistent with previous findings [67]. At a national level (in India), the model had predicted that 15% of the total area would be suitable for coconut cultivation under the current climate with the climate being placed in the high suitability category all along the west coast (Karnataka and Kerala), in the moderate suitability category in some parts of South Interior Karnataka and TN, while the rest of the south interior would be placed in the low suitability category and the eastern region (TN, AP, Odisha, West Bengal) and Northeastern states would be placed in the low to very low suitability category, which is in agreement with the actual spread of coconut as in Figure 1a.

Previous studies had concluded that global warming had greatly influenced the climate of the region, thereby causing expansions, shifts, or contractions in the area under cultivation [72,73]. From Figure 9, it is clear that the majority of the area expansion is happening in the barely suitable category, which has <0.25 value of the maximum training sensitivity plus specificity and is, therefore, not taken into account for suitable area calculation. Our predictions showed that under the future climate, at a national level (in India), the total area suitable for coconut cultivation would remain constant (15%). However, at the regional level, the areas with a potentially suitable climate for coconut will shift from different categories like low, moderate, and high suitability. The west coast will be placed in the high suitability category, the south interior will be placed in the moderate to high suitability category, and the east coast will be placed in the low suitability category for coconut cultivation. These results were consistent with previous studies showing that the habitat suitability of plant species was predicted to become vulnerable in some regions under climate change conditions [coffee could be replaced by cocoa in Mesoamerica [74], cocoa in Latin America [75], cocoa in West Africa [34], coffee in Zimbabwe [76], and *C. tinctorius* in China [73].

Even under high concentration scenarios, the model projection of high climate suitability for coconut all along the west coast showed a high probability of its cultivation. In the south interior, the climate of some of the current areas of high probable occurrence may change to moderate suitability, moderate to low suitability, and low suitability to unsuitable. In the eastern region, the climate is less suitable, and the areas with a suitable climate were found towards the west, especially in TN. Small changes in the temperature and precipitation seasonality could be the reason for low climate vulnerability to climate change for coconut in the west coast. The south interior regions are low rainfall areas, even though the predicted precipitation is less variable, still, the temperature-induced rise in evapotranspiration might subject the plants to drought in areas without irrigation facilities. This is further exasperated by the prevailing low humidity during the summer months. High temperature under low humidity is more detrimental to fertilization [23]. In the eastern region, where the summer temperature is already high (T_max_ > 40 °C), the projected temperature rise of 2 °C to 3 °C would make the crop more vulnerable. These regions showed high variability in temperature (Bio 4) and precipitation seasonality (Bio 15) from the current climate. Therefore, the proposed contraction to the area with a suitable will be a potential threat to the increasing demand for coconut and coconut products worldwide. The market demand of coconut is rather great in India due to the rich nutrients and oil provided the coconut, as well as the latest discoveries of its nutraceutical benefits [9].

The suitable area available for the cultivation of coconut will gradually decrease because of urban development and other social causes. Furthermore, there is more area becoming vulnerable under the future climate. Together, these factors add pressure to produce more from each unit land area to meet the growing demand. Therefore, more attention and additional protective measures should be given to extensive coconut cultivation areas in the south interior regions so as to ensure the sustainable cultivation of coconut, at least in presently cultivated areas. In these regions, high temperatures and low humidity are the major problem. The effect of high temperatures, to a certain extent, could be alleviated either by planting genotypes with wider adaptability for water deficit and high temperatures or by adopting some of the agro techniques of fertigation along with soil moisture conservation practices like mulching, bunding, and cropping systems [77]. Soil moisture retention, summer irrigation, drip irrigation, and fertilizer application are only a few of the agronomic adaptations that can not only reduce losses but also boost productivity in the majority of coconut-growing areas because the availability of water to the palms can help them in canopy cooling by transpiration and can partially offset the adverse influences of high temperatures [67]. In the eastern regions, where T_max_ is the major issue, adopting genotypes with wider adaptability to high temperatures may sustain the crop under the future climate.

## 5. Conclusions

Coconut, an important plantation crop grown in coastal belts and plains, is highly vulnerable to climate change, and there is urgent need for its appropriate protection and management. In this study, we used the MaxEnt model to evaluate the bioclimatic variables determining the suitable habitat for coconut cultivation and to predict the regions with a climate that is potentially suitable under the future climate conditions. The MaxEnt model is popularly used for species-distribution due its prediction accuracy even with incomplete data and a small sample size, but it has some limitations, such as underestimating the influence of parameters and spatial bias in the occurrence data and the possibility of overfitting and the lack of its capacity to generalize results from independent data. In addition, the MaxEnt outputs gives environmental suitability rather than predicted probability of occurrence. Considering that we have limited data on coconut, the MaxEnt model was used to obtain an overall understanding of the suitability in different regions of India. Our results have shown that coconut will contract its suitable climate area size and will face a high risk of unfavorable climate in the southern interior and eastern regions of India in response to global climate change. There is shift in climate suitability from high to moderate, moderate to low and low to unsuitable under the future climate. Effective coordination amongst all stakeholders is essential to develop and implement adaptation strategies so as to ensure sustainable cultivation of coconut at least in presently cultivated areas. Future research should be focused on collecting robust data at more granular and complete datasets and use them for modeling the impacts of climate change and their impact on crop yield with better mechanistic models. In addition, using an ensemble of models rather than one single model will improve accuracy of predictions.

## Figures and Tables

**Table 1:** Characteristics of some of the major coconut growing districts of India from which the occurrence data was collected as input to MaxEnt model. Humidity is the range shown for the summer months (March, April, and May). Occurrence points are the number of points for each location after model rarefaction.

State	Area (000 ha)	Major Districts	Area (000 ha)	Latitude	Longitude	Soil Type	Temperature Range (°C)		Humidity (%) (Range)	Precipitation (mm)	Occurrence Points
							Minimum	Maximum			
Kerala	761	Kozhikode	112.80	11.2588° N	75.7804° E	Alluvial, lateritic	16.9–24	28.2–36	46–92	3592	51
		Malappuram	105.09	11.0510° N	76.0711° E	Loamy	12–24.2	23.7–36	44–94	2877	60
		Kannur	85.97	11.8745° N	75.3704° E	Sandy loam to clay	18.1–23.8	30–36	45–95	3831	51
		Thrissur	80.58	12.4996° N	74.9869° E	Sandy loam to sandy	17–24.2	29.8–34	53–99	3162	44
		Kasaragod	67.08	12.4996° N	74.9869° E	Red sandy loam, sandy	20.2–23.8	31.5–36	36–93	4245	38
Karnataka	619	Tumkur	157.37	13.3379° N	77.1173° E	Red loamy & black	15–17.4	31–37.1	11–90	554	89
		Hassan	52.32	13.0033° N	76.1004° E	-do-	14.8–17.7	30–36	34–94	1276	36
		Chikkmagaluru	40.93	13.3161° N	75.7720° E	Clay loam	11.8–18.3	28–36	33–93	2019	28
		Chitradurga	40.80	14.2251° N	76.3980° E	Red sandy loam	15.7–17.8	33.9–38	11–90	508	27
		D. Kannada	20.39	12.8438° N	75.2479° E	Laterite & sandy loam	13.9–23.2	28–35.2	36–93	4089	10
Tamil Nadu	436	Coimbatore	87.41	11.0168° N	76.9558° E	Sandy loam	10.2–20.9	23.7–38	23–95	1149	48
		Thiruppur	60.33	11.1085° N	77.3411° E	Loamy & alluvial	10.2–20.9	23.7–38	23–95	1149	30
		Thanjavur	37.33	10.7870° N	79.1378° E	Sandy	21.1–23.3	34.4–41	30–91	850	24
		Dindigul	29.22	10.3624° N	77.9695° E	Loamy & Sandy loam	8.6–20.5	22.5–41	29–90	1015	25
		Kanyakumari	24.10	8.0883° N	77.5385° E	Saline & Coastal	16.4–24.2	25.7–36	47–95	1254	15
Andhra Pradesh	111	E. Godavari	52.30	17.3213° N	82.0407° E	Red clay & alluvial	13–22.2	31.3–42	30–93	1274	12
		W. Godavari	22.09	16.9174° N	81.3399° E	Alluvial & sandy alluvial	15.6–21.3	34.5–43	49–98	1166	36

**Table 2:** Bioclimatic and topographic variables (in bold texts) used for predicting habitat suitability for coconut in India and their contribution to habitat suitability.

Category Units	Sources	Variables	Abbreviations	Units	% Contribution
Bioclimatic	WorldClim—Global Climate Data http://www.worldclim.org/, accessed on 22 May 2020 Paleoclim.org	**Annual mean temperature**	**BIO1**	°C	1.0
		**Mean diurnal range**	**BIO2**	°C	3.1
		**Isothermality**	**BIO3**	unitless	5.6
		**Temperature seasonality**	**BIO4**	unitless	34.4
		Max. temperature of warmest month	BIO5	°C	
		**Min. temperature of coldest month**	**BIO6**	°C	4.4
		**Temperature annual range**	**BIO7**	°C	28.7
		Mean temp of wettest quarter	BIO8	°C	
		Mean temp of driest quarter	BIO9	°C	
		Mean temp of warmest quarter	BIO10	°C	
		Mean temp of coldest quarter	BIO11	°C	
		**Annual precipitation**	**BIO12**	mm	2.0
		Precipitation of wettest month	BIO13	mm	
		**Precipitation of driest month**	**BIO14**	mm	4.2
		**Precipitation seasonality**	**BIO15**	unitless	8.6
		Precipitation of wettest quarter	BIO16	mm	
		Precipitation of driest quarter	BIO17	mm	
		**Precipitation of warmest quarter**	**BIO18**	mm	1.1
		**Precipitation of coldest quarter**	**BIO19**	mm	2.2
Topographical	http://www.fao.org/soils, accessed on 22 May 2020	**Soil**		unitless	0.4
	http://due.esrin.esa.int/page_globcover.php, accessed on 22 May 2020	**Land cover**		unitless	0.7
	https://cgiarcsi.community/data/srtm, accessed on 22 May 2020	**Elevation**		meter	3.5

**Table 3:** Correlation coefficients (Rs) between 19 environmental variables, 2 topographical variables (elevation, land cover) and soil using the SDM Tool box 2.0.

Layer	Bio 1	Bio 2	Bio 3	Bio 4	Bio 5	Bio 6	Bio 7	Bio 8	Bio 9	Bio 10	Bio 11	Bio 12	Bio 13	Bio 14	Bio 15	Bio 16	Bio 17	Bio 18	Bio 19	Soil	Land Cover	Elevation
Bio 1	1.000																					
Bio 2	0.178	1.000																				
Bio 3	0.359	0.117	1.000																			
Bio 4	−0.322	0.325	−0.798	1.000																		
Bio 5	0.848	0.620	0.257	−0.075	1.000																	
Bio 6	0.820	−0.197	0.649	−0.761	0.537	1.000																
Bio 7	−0.051	0.827	−0.450	0.751	0.405	−0.553	1.000															
Bio 8	0.787	0.137	−0.120	0.190	0.622	0.396	0.185	1.000														
Bio 9	**0.864**	0.117	0.651	−0.614	0.707	**0.895**	−0.272	0.480	1.000													
Bio 10	**0.936**	0.397	0.173	−0.058	**0.944**	0.631	0.248	0.782	0.739	1.000												
Bio 11	**0.886**	0.026	0.657	−0.706	0.698	**0.968**	−0.359	0.454	**0.939**	0.741	1.000											
Bio 12	−0.362	−0.563	−0.277	−0.021	−0.569	−0.207	−0.338	−0.199	−0.368	−0.464	−0.303	1.000										
Bio 13	−0.226	−0.433	−0.119	−0.167	−0.389	−0.067	−0.311	−0.167	−0.172	−0.335	−0.126	**0.913**	1.000									
Bio 14	−0.303	−0.529	−0.040	−0.038	−0.524	−0.091	−0.419	−0.218	−0.300	−0.403	−0.237	0.289	0.041	1.000								
Bio 15	0.183	0.393	−0.085	0.131	0.338	−0.056	0.395	0.253	0.167	0.264	0.067	0.016	0.315	−0.560	1.000							
Bio 16	−0.249	−0.464	−0.163	−0.128	−0.419	−0.094	−0.311	−0.171	−0.213	−0.351	−0.162	**0.946**	**0.993**	0.090	0.256	1.000						
Bio 17	−0.309	−0.556	−0.164	0.056	−0.536	−0.149	−0.367	−0.156	−0.363	−0.398	−0.296	0.343	0.062	**0.948**	−0.582	0.120	1.000					
Bio 18	−0.537	−0.371	−0.457	0.389	−0.664	−0.528	−0.084	−0.156	−0.613	−0.543	−0.613	0.666	0.393	0.400	−0.265	0.456	0.447	1.000				
Bio 19	0.001	−0.339	0.262	−0.395	−0.162	0.244	−0.425	−0.149	0.148	−0.142	0.178	0.452	0.541	0.039	0.094	0.535	0.017	−0.039	1.000			
Soil	−0.361	−0.158	−0.302	0.242	−0.315	−0.318	0.034	−0.205	−0.383	−0.304	−0.376	0.083	0.030	0.218	−0.103	0.039	0.257	0.099	−0.043	1.000		
Land Cover	−0.267	−0.086	−0.147	0.151	−0.290	−0.269	0.004	−0.118	−0.270	−0.277	−0.290	0.237	0.167	0.160	0.046	0.182	0.163	0.266	0.102	0.061	1.000	
Elevation	−0.809	0.051	0.017	0.032	−0.555	−0.537	0.034	**−0.902**	−0.556	−0.727	−0.563	0.039	−0.003	0.108	−0.197	0.002	0.056	0.165	−0.005	0.239	0.128	1.000

**Table 4:** Generated and selected candidate models and their fit and validation statistics.

Criteria	Number_of_Models
All candidate models	2480
Statistically significant models	2480
Models meeting omission rate criteria	0
Models meeting AICc criteria	2
Statistically significant models meeting omission rate criteria	0
Statistically significant models meeting AICc criteria	2
Statistically significant models meeting omission rate and AICc criteria	0
Selected model	M_0.1_F_qp
Statistics of the selected model	
Mean AUC ratio	1.501
Rate of omission > 0.05%	0.057
AICc	18,636.44
Delta AICc	0

**Table 5:** Annual mean temperature (Bio 1), Isothermality (Bio 3), Temperature Seasonality (Bio 4), Temperature Annual Range (Bio 7), and Precipitation Seasonality (Bio 15) for the current and future scenarios of the 2050s and 2070s under RCP 4.5 and RCP 8.5 of India and major coconut growing states. The values in brackets indicate change from the current value.

Bio Variable	Country/State	Current	RCP 4.5		RCP 8.5	
			2050s	2070s	2050s	2070s
Bio 1 (°C)	India	25.64	27.37 (1.73)	27.78 (2.14)	27.85 (2.21)	28.94 (3.3)
	Andhra Pradesh (AP)	27.37	29.15 (1.77)	29.54 (2.16)	29.41 (2.04)	30.51 (3.14)
	Karnataka	25.55	27.05 (1.49)	27.40 (1.84)	27.53 (1.97)	28.64 (3.08)
	Tamil Nadu (TN)	27.26	28.96 (1.70)	29.30 (2.04)	29.43 (2.17)	30.44 (3.18)
	Kerala	26.13	27.59 (1.46)	27.94 (1.81)	28.08 (1.95)	29.00 (2.86)
Bio 3 (%)	India	40.79	47.44 (6.65)	47.64 (6.85)	47.60 (6.81)	47.30 (6.51)
	AP	42.33	46.79 (4.46)	47.32 (4.99)	46.92 (4.59)	46.39 (4.06)
	Karnataka	45.32	53.18 (7.86)	53.28 (7.96)	53.21 (7.89)	52.49 (7.17)
	TN	48.97	56.03 (7.06)	56.61 (7.64)	56.18 (7.21)	54.77 (5.80)
	Kerala	47.61	61.63 (14.02)	62.12 (14.51)	62.25 (14.64)	62.17 (14.56)
Bio 4 (%)	India	29.79	31.37 (1.58)	30.93 (1.14)	31.1 (1.31)	31.34 (1.55)
	AP	26.27	29.48 (3.21)	28.89 (2.62)	28.62 (2.35)	29.66 (3.39)
	Karnataka	20.85	21.96 (1.11)	21.84 (0.99)	22.27 (1.42)	23.39 (2.54)
	TN	19.37	21.39 (2.02)	20.83 (1.46)	21.3 (1.93)	22.28 (2.91)
	Kerala	10.76	11.74 (0.98)	11.64 (0.88)	11.87 (1.11)	12.35 (1.59)
Bio 7 (°C)	India	20.09	22.05 (1.96)	22.03 (1.91)	21.85 (1.76)	21.87 (1.78)
	AP	18.12	20.50 (2.38)	20.26 (2.14)	19.86 (1.75)	20.12 (2.00)
	Karnataka	18.20	19.45 (1.26)	19.27 (1.08)	19.33 (1.14)	19.56 (1.37)
	TN	14.64	16.10 (1.45)	15.80 (1.16)	15.96 (1.32)	16.42 (1.77)
	Kerala	10.72	11.88 (1.16)	11.65 (0.94)	11.71 (0.99)	11.86 (1.14)
Bio 15 (%)	India	103.8	106.3 (2.5)	104.3 (0.5)	105.6 (1.8)	107.7 (3.9)
	AP	88.59	92.54 (3.95)	91.45 (2.86)	92.14 (3.55)	94.41 (5.82)
	Karnataka	98.44	101.39 (2.95)	99.41 (0.97)	100.55 (2.11)	103.38 (4.94)
	TN	76.81	83.24 (6.43)	79.65 (2.84)	81.5 (4.69)	84.65 (7.84)
	Kerala	90.35	87.02 (−3.33)	83.02 (−7.33)	85.79 (−4.56)	87.01 (−3.34)

**Table 6:** Climatically suitable and unsuitable areas for growing coconut under the current and the future climate of RCP 4.5 and RCP 8.5 and the range expansion and contraction (the values in brackets are the difference in percentage from total study area) as modeled by MaxEnt for India and major coconut growing states.

Country/State	Category	Area (km^2^)				
		Current	RCP 4.5		RCP 8.5	
			2050	2070	2050	2070
India (Study area)	Range expansion		52,617 (3.8%)	61,642 (4.4%)	75,383 (5.4%)	65,164 (4.7%)
	Unsuitable	1,184,077 (85%)	1,131,238 (81.2%)	1,122,212 (80.5%)	1,108,471 (79.6%)	1,118,691 (80.3%)
	Suitable	209,374 (15%)	161,776 (11.6%)	168,738 (12.1%)	173,169 (12.4%)	172,036 (12%)
	Range contraction		47,578 (3.4%)	40,616 (2.9%)	36,184 (2.6%)	37,317 (2.7%)
Karnataka	Range expansion		27,956 (14.5%)	28,348 (14.7%)	38,858 (20.2%)	24,880 (12.9%)
	Unsuitable	11,4714 (60)	86,759 (45.1%)	86,366 (44.9%)	75,857 (39.4%)	89,835 (46.7%)
	Suitable	77,591 (40)	60,723 (31.6%)	62,994 (32.8%)	67,846 (35.3%)	63,448 (33.0%)
	Range contraction		16,869 (8.8%)	14,597 (7.6%)	9745 (5.1%)	14,143 (7.4%)
Kerala	Range expansion		1051 (2.8%)	798 (2.2%)	1009 (2.7%)	1807 (4.9%)
	Unsuitable	7439 (20%)	6388 (17.3%)	6640 (17.9%)	6430 (17.4%)	5631 (15.2%)
	Suitable	29,565 (80%)	28,599 (77.3%)	28,641 (77.4%)	28,536 (77.1%)	28,998 (78.4%)
	Range contraction		967 (2.6%)	925 (2.5%)	1030 (2.8%)	567 (1.5%)
Tamil Nadu	Range expansion		9804 (7.5%)	15,346 (11.8%)	15,283 (11.7%)	16,144 (12.4%)
	Unsuitable	54,141 (42%)	44,337 (34.0%)	38,795 (29.7%)	38,858 (29.8%)	37,998 (29.1%)
	Suitable	76,289 (58%)	67,997 (52.1%)	71,356 (54.7%)	71,041 (54.5%)	72,531 (55.6%)
	Range contraction		8292 (6.4%)	4933 (3.8%)	5248 (4.0%)	3758 (2.9%)

## Data Availability

Data is available upon request from the corresponding author.

## References

[1] APCC (2015). Coconut Statistical Yearbook.

[2] Omont H. (2001). Information Sheet-COCONUT.

[3] Rethinam P., Nampoothiri K.U.K., Krishnakumar V., Thampan P.K., Nair M.A. (2018). International Scenario of Coconut Sector. The Coconut Palm (Cocos nucifera L.)—Research and Development Perspectives.

[4] Ramesh S.V., Pandiselvam R., Thushara R., Manikantan M.R., Hebbar K.B., Beegum S., Mathew A.C., Neenu S., Shil S. (2020). Engineering intervention for production of virgin coconut oil by hot process and multivariate analysis of quality attributes of virgin coconut oil extracted by various methods. J. Food Process Eng..

[5] Hebbar K.B., Arivalagan M., Manikantan M.R., Mathew A.C., Thamban C., Thomas G.V., Chowdappa P. (2015). Coconut inflorescence sap and its value addition as sugar—Collection techniques, yield, properties and market perspective. Curr. Sci..

[6] Hebbar K.B., Arivalagan M., Pavithra K.C., Roy T.K., Gopal M., Shivashankara K.S., Chowdappa P. (2020). Nutritional profiling of coconut (*Cocos nucifera* L.) inflorescence sap collected using novel coco-sap chiller method and its value added products. J. Food Meas. Charact..

[7] Asghar M.T., Yusof Y.A., Mokhtar M.N., Ya’acob M.E., Mohd. Ghazali H., Chang L.S., Manaf Y.N. (2020). Coconut (*Cocos nucifera* L.) sap as a potential source of sugar: Antioxidant and nutritional properties. Food Sci. Nutr..

[8] Joshi S., Kaushik V., Gode V., Mhaskar S. (2020). Coconut Oil and Immunity: What do we really know about it so far?. J. Assoc. Phys. India.

[9] Ramesh S.V., Krishnan V., Praveen S., Hebbar K.B. (2021). Dietary prospects of coconut oil for the prevention and treatment of Alzheimer’s disease (AD): A review of recent evidences. Trends Food Sci. Technol..

[10] Jose T.K. (2015). Let’s ‘Make in India’ futuristic coconut products. Indian Coconut J..

[11] OECD-FAO (2017). Agricultural Outlook 2017–2026.

[12] Pachauri R.K., Allen M.R., Barros V.R., Broome J., Cramer W., Christ R., Church J.A., Clarke L., Dahe Q., Dasgupta P. (2014). Climate Change 2014: Synthesis Report. Contribution of Working Groups I, II and III to the Fifth Assessment Report of the Intergovernmental Panel on Climate Change.

[13] Kumar S.N., Bai K.V.K., Rajagopal V., Aggarwal P.K. (2009). Simulating coconut growth, development and yield with the InfoCrop-coconut model (Tree Physiology 28 (1049–1058)). Tree Physiol..

[14] Wilson C.D., Roberts D., Reid N. (2011). Applying species distribution modelling to identify areas of high conservation value for endangered species: A case study using *Margaritifera* (L.). Biol. Conserv..

[15] Hebbar K.B., Santhosh A., Sukumar A.P., Neethu P., Ramesh S.V., Selvamani V. (2021). Effect of sea water substitution on growth, physiological and biochemical processes of coconut (*Cocos nucifera* L.) seedlings—A hydroponic study. Sci. Hortic. (Amst.).

[16] Walther G.R., Post E., Convey P., Menzel A., Parmesan C., Beebee T.J.C., Fromentin J.M., Hoegh-Guldberg O., Bairlein F. (2002). Ecological responses to recent climate change. Nature.

[17] Parmesan C., Yohe G. (2003). A globally coherent fingerprint of climate change impacts across natural systems. Nature.

[18] Bellard C., Bertelsmeier C., Leadley P., Thuiller W., Courchamp F. (2012). Impacts of climate change on the future of biodiversity. Ecol. Lett..

[19] Staudinger M.D., Grimm N.B., Staudt A., Carter S.F., Chapin F.S., Kareiva P., Ruckelshaus M., Stein B.A. (2012). Impacts of climate change on biodiversity, ecosystems, and ecosystem services: Technical input to the 2013 National Climate Assessment. Cooperative Report to the 2013 National Climate Assessment.

[20] Hebbar K.B., Sheena T.L., Kumari K.S., Padmanabhan S., Balasimha D., Kumar M., Thomas G.V. (2013). Response of coconut seedlings to elevated CO_2_ and high temperature in drought and high nutrient conditions. J. Plant. Crop..

[21] Norby R.J., DeLucia E.H., Gielen B., Calfapietra C., Giardina C.P., Kings J.S., Ledford J., McCarthy H.R., Moore D.J.P., Ceulemans R. (2005). Forest response to elevated CO_2_ is conserved across a broad range of productivity. Proc. Natl. Acad. Sci. USA.

[22] Hebbar K.B., Rose H.M., Nair A.R., Kannan S., Niral V., Arivalagan M., Gupta A., Samsudeen K., Chandran K.P., Chowdappa P. (2018). Differences in In Vitro Pollen Germination and Pollen tube Growth of Coconut (Cocos nucifera L.) Cultivars in Response to High Temperature Stress.

[23] Hebbar K.B., Neethu P., Sukumar P.A., Sujithra M., Santhosh A., Ramesh S.V., Niral V., Hareesh G.S., Nameer P.O., Prasad P.V.V. (2020). Understanding physiology and impacts of high temperature stress on the progamic phase of coconut (*Cocos nucifera* L.). Plants.

[24] Oren R., Ellsworth D.S., Johnsen K.H., Phillips N., Ewers B.E., Maier C., Schäfer K.V.R., McCarthy H., Hendrey G., McNulty S.G. (2001). Soil fertility limits carbon sequestration by forest ecosystems in a CO_2_-enriched atmosphere. Nature.

[25] Reich P.B., Luo Y., Bradford J.B., Poorter H., Perry C.H., Oleksyn J. (2014). Temperature drives global patterns in forest biomass distribution in leaves, stems, and roots. Proc. Natl. Acad. Sci. USA.

[26] Hebbar K.B., Apshara E., Chandran K.P., Prasad P.V.V. (2020). Effect of elevated CO_2_, high temperature, and water deficit on growth, photosynthesis, and whole plant water use efficiency of cocoa (*Theobroma cacao* L.). Int. J. Biometeorol..

[27] Phillips S.J., Dudík M., Schapire R.E., Internet Maxent Software for Modeling Species Niches and Distributions (Version 3.4.1). http://biodiversityinformatics.amnh.org/open_source/maxent/.

[28] Elith J., Graham C.H., Anderson R.P., Dudík M., Ferrier S., Guisan A., Hijmans R.J., Huettmann F., Leathwick J.R., Lehmann A. (2006). Novel methods improve prediction of species’ distributions from occurrence data. Ecography.

[29] Pearson R.G., Dawson T.P. (2003). Predicting the impacts of climate change on the distribution of species: Are bioclimate envelope models useful?. Glob. Ecol. Biogeogr..

[30] Braunisch V., Coppes J., Arlettaz R., Suchant R., Schmid H., Bollmann K. (2013). Selecting from correlated climate variables: A major source of uncertainty for predicting species distributions under climate change. Ecography.

[31] Yang X.-Q., Kushwaha S.P.S., Saran S., Xi J., Roy P.S. (2013). Maxent modeling for predicting the potential distribution of medicinal plant, *Justicia adhatoda* L. in Lesser Himalayan foothill. Ecol. Eng..

[32] Searcy C.A., Shaffer H.B. (2016). Do ecological niche models accurately identify climatic determinants of species ranges?. Am. Nat..

[33] Läderach P., Martinez-Valle A., Schroth G., Castro N. (2013). Predicting the future climatic suitability for cocoa farming of the world’s leading producer countries, Ghana and Côte d’Ivoire. Clim. Chang..

[34] Schroth G., Läderach P., Martinez-Valle A.I., Bunn C., Jassogne L. (2016). Vulnerability to climate change of cocoa in West Africa: Patterns, opportunities and limits to adaptation. Sci. Total Environ..

[35] Pham Y., Reardon-Smith K., Mushtaq S., Cockfield G. (2019). The impact of climate change and variability on coffee production: A systematic review. Clim. Chang..

[36] Kogo B.K., Kumar L., Koech R., Kariyawasam C.S. (2019). Modelling climate suitability for rainfed Maize cultivation in Kenya using a Maximum Entropy (MaxENT) approach. Agronomy.

[37] He Q., Zhou G. (2016). Climate-associated distribution of summer maize in China from 1961 to 2010. Agric. Ecosyst. Environ..

[38] Jayasinghe S.L., Kumar L. (2019). Modeling the climate suitability of tea [*Camellia sinensis* (L.) O. Kuntze] in Sri Lanka in response to current and future climate change scenarios. Agric. For. Meteorol..

[39] Cobos M.E., Townsend Peterson A., Barve N., Osorio-Olvera L. (2019). Kuenm: An R package for detailed development of ecological niche models using Maxent. PeerJ.

[40] Brown J.L., Bennett J.R., French C.M. (2017). SDMtoolbox 2.0: The next generation Python-based GIS toolkit for landscape genetic, biogeographic and species distribution model analyses. PeerJ.

[41] Brown J.L., Hill D.J., Dolan A.M., Carnaval A.C., Haywood A.M. (2018). Paleoclim, high spatial resolution paleoclimate surfaces for global land areas. Sci. Data.

[42] Hijmans R.J., Cameron S.E., Parra J.L., Jones P.G., Jarvis A. (2005). Very high resolution interpolated climate surfaces for global land areas. Int. J. Climatol..

[43] Remya K., Ramachandran A., Jayakumar S. (2015). Predicting the current and future suitable habitat distribution of *Myristicadactyloides* Gaertn. Using MaxEnt model in the Eastern Ghats, India. Ecol. Eng..

[44] Jose V.S., Nameer P.O. (2020). The expanding distribution of the Indian Peafowl (*Pavocristatus*) as an indicator of changing climate in Kerala, southern India: A modelling study using MaxEnt. Ecol. Indic..

[45] Mod H.K., Scherrer D., Luoto M., Guisan A. (2016). What we use is not what we know: Environmental predictors in plant distribution models. J. Veg. Sci..

[46] Shabani F., Ahmadi M., Kumar L., Solhjouy-Fard S., Tehrany M.S., Shabani F., Kalantar B., Esmaeili A. (2020). Invasive weed species’ threats to global biodiversity: Future scenarios of changes in the number of invasive species in a changing climate. Ecol. Indic..

[47] Jaynes E.T. (1957). Information Theory and Statistical Mechanics. Phys. Rev..

[48] Phillips S.J., Anderson R.P., Schapire R.E. (2006). Maximum entropy modeling of species geographic distributions. Ecol. Model..

[49] Phillips S.J., Dudík M. (2008). Modeling of species distribution with MaxEnt: New extensions and a comprehensive evaluation. Ecography.

[50] O’Donnell M.S., Ignizio D.A. (2012). Bioclimatic Predictors for Supporting Ecological Applications in the Conterminous United States. US Geol. Surv. Data Ser..

[51] Merow C., Smith M.J., Silander J.A. (2013). A practical guide to MaxEnt for modeling species’ distributions: What it does, and why inputs and settings matter. Ecography.

[52] Phillips S.J., Dudík M., Schapire R.E. (2004). A maximum entropy approach to species distribution modeling. Proceedings of the Twenty-First International Conference on Machine Learning.

[53] Al Ruheili A.M., Boluwade A., Al Subhi A.M. (2021). Assessing the impact of climate change on the distribution of lime (16SRII-b) and alfalfa (16srii-d) phytoplasma disease using maxent. Plants.

[54] Liu C., Newell G., White M. (2016). On the selection of thresholds for predicting species occurrence with presence-only data. Ecol. Evol..

[55] Davies T.J., Purvis A., Gittleman J.L. (2009). Quaternary climate change and the geographic ranges of mammals. Am. Nat..

[56] Zhang L., Cao B., Bai C., Li G., Mao M. (2016). Predicting suitable cultivation regions of medicinal plants with Maxent modeling and fuzzy logics: A case study of *Scutellaria baicalensis* in China. Environ. Earth Sci..

[57] Xu X., Zhang H., Yue J., Xie T., Xu Y., Tian Y. (2018). Predicting shifts in the suitable climatic distribution of walnut (*Juglans regia* L.) in China: Maximum entropy model paves the way to forest management. Forests.

[58] Kelly A.E., Goulden M.L. (2008). Rapid shifts in plant distribution with recent climate change. Proc. Natl. Acad. Sci. USA.

[59] Blach-Overgaard A., Svenning J.-C., Balslev H. (2009). Climate change sensitivity of the African ivory nut palm, *Hyphaene petersiana* Klotzsch ex Mart. (Arecaceae)—A keystone species in SE Africa. IOP Conference Series: Earth and Environmental Science.

[60] Blach-Overgaard A., Svenning J.C., Dransfield J., Greve M., Balslev H. (2010). Determinants of palm species distributions across Africa: The relative roles of climate, non-climatic environmental factors, and spatial constraints. Ecography.

[61] Lieberman D., Lieberman M., Peralta R., Hartshorn G.S. (1996). Tropical forest structure and composition on a large-scale altitudinal gradient in Costa Rica. J. Ecol..

[62] Walther G.R., Gritti E.S., Berger S., Hickler T., Tang Z.Y., Sykes M.T. (2007). Palms tracking climate change. Glob. Ecol..

[63] Zuquim G., Costa F.R.C., Tuomisto H., Moulatlet G.M., Figueiredo F.O.G. (2020). The importance of soils in predicting the future of plant habitat suitability in a tropical forest. Plant Soil.

[64] Child R. (1974). Coconuts.

[65] Persley G.J. (1992). Replanting the Tree of Life towards an International Agenda for Coconut Palm Research.

[66] Rajagopal V., Parthasarathy V.A., Kumar S.N., Reddy D.V.S., Rohini I., Parthasarathy V.A., Chattopadyay P.K., Bose T.K. (2006). Coconut.

[67] Kumar S.N., Aggarwal P.K. (2013). Climate change and coconut plantations in India Impacts and potential adaptation gains. Agric. Syst..

[68] Hebbar K.B., Berwal M.K., Chaturvedi V.K. (2016). Plantation crops: Climatic risks and adaptation strategies. Indian J. Plant Physiol..

[69] Ranasinghe C.S., Silva L.R.S., Premasiri R.D.N. (2015). Major determinants of fruit set and yield fluctuation in coconut (*Cocos nucifera* L.). J. Natl. Sci. Found. Sri Lanka.

[70] Samanta M.K., Chattopadhyay N., Hore J.K., Alam K. (2013). Associationship of weather parameters on the Floral characteristics of coconut. Acta Hortic..

[71] Samarasinghe C.R.K., Meegahakumbura M.K., Dissanayaka H.D.M.A.C., Kumarathunge D., Perera L. (2018). Variation in yield and yield components of different coconut cultivars in response to within year rainfall and temperature variation. Sci. Hortic. (Amst.).

[72] Thomas C.D., Cameron A., Green R.E., Bakkenes M., Beaumont L.J., Collingham Y.C., Erasmus B.F.N., De Siqueira M.F., Grainger A., Hannah L. (2004). Letter to nature: Extinction risk from climate change. Nature.

[73] Wei B., Wang R., Hou K., Wang X., Wu W. (2018). Predicting the current and future cultivation regions of Carthamus tinctorius L. using MaxEnt model under climate change in China. Glob. Ecol. Conserv..

[74] de Sousa K., van Zonneveld M., Holmgren M., Kindt R., Ordoñez J.C. (2019). The future of coffee and cocoa agroforestry in a warmer Mesoamerica. Sci. Rep..

[75] Fernandez-Manjarrés J. (2019). Using ecological modelling tools to inform policy makers of potential changes in crop distribution: An example with cacao crops in Latin America. Economics Tools Methods Analysis of Global Change Impacts on Agriculture and Food Security.

[76] Chemura A., Kutywayo D., Chidoko P., Mahoya C. (2016). Bioclimatic modelling of current and projected climatic suitability of coffee (Coffea arabica) production in Zimbabwe. Reg. Environ. Chang..

[77] Subramanian P., Dhanapal R., Mathew A.C., Palaniswami C., Upadhyaya A.K., Naresh Kumar S., Reddy D.V. (2012). Effect of fertilizer application through micro-irrigation technique on nutrient availability and coconut productivity. J. Plant. Crops.

